# IgA Monoclonal Gammopathy of Undetermined Significance and Complication of *Streptococcus mitis* Bacteremia

**DOI:** 10.1155/2020/8823908

**Published:** 2020-10-10

**Authors:** Jennifer Paterno, Alessandra Petrillo, David Samuel Dicapua Siegel, Chinwe Ogedegbe

**Affiliations:** Hackensack University Medical Center, 30 Prospect Ave, Hackensack, NJ, USA

## Abstract

This case presents a patient with bacteremia of an unusual organism with a history of monoclonal gammopathy of undetermined significance (MGUS). MGUS is typically thought to be asymptomatic until potential progression of the disease. This case reports a patient with a history of MGUS who does not show disease progression, however, may be showing symptoms, such as immunodeficiency. This case displays bacteremia with *Streptococcus mitis* within a two-week period of an invasive procedure. Recent studies parallel this case by showing MGUS patients may have two times the risk of infections compared to the unaffected population. This report brings up the question of taking prophylactic measures for this patient population to prevent future complications.

## 1. Introduction

Monoclonal gammopathy of undetermined significance (MGUS) is a disorder in which there is an increased production and accumulation of an abnormal protein, monoclonal protein (M protein), by plasma cells in the bone marrow [1]. There are two subtypes of this disease, IgM MGUS and non-IgM MGUS. Non-IgM MGUS can be further broken down into the following immunoglobulin (Ig) classes: IgG, IgA, IgD, and light chain MGUS [2]. While MGUS itself is considered a benign and asymptomatic disease, it has the potential to progress to Waldenström's macroglobulinemia (WM), multiple myeloma (MM), or B-cell neoplasms including chronic lymphocytic leukemia (CLL) and marginal zone lymphoma (MZL) [1]. Three risk factors are identified as a tool to further assess an individual's probability of disease progression which include serum M protein greater than or equal to 15 g/L, criteria for non-IgM MGUS, and an abnormal serum free light chain ratio [2]. A patient is classified as low risk if they have zero risk factors, low-intermediate risk with one risk factor, intermediate-high risk if they have two risk factors, and high risk with three risk factors [2].

Diagnosis of MGUS is confirmed with an abnormal protein electrophoresis showing the presence of M protein. Criteria for diagnosis are met if serum M protein is less than 30 g/L, bone marrow clonal plasma cells are less than 10%, and there is an absence of plasma cell myeloma related end-organ damage. Signs of end-organ damage include hypercalcemia, anemia, renal insufficiency, and bone lesions [2]. There is no treatment currently for MGUS patients. However, close follow up is recommended to monitor disease progression and complications. Possible complications of MGUS include neuropathy, osteoporosis, and infection [1]. While neuropathy and osteoporosis may be the consequence of production of abnormal immunoglobulins or other factors produced by these clones, infection is the fundamental result of the immunodeficient state. This suppression of immunity in MGUS is secondary to a global decrease in production of all immunoglobulin isotypes, regardless of the subtype of disease.

Immunoglobulin A (IgA) is an important antibody involved with mucosal immunity. IgA is the most bountiful immunoglobulin present in mucosal secretions and plays an important role in maintenance of commensal microorganisms [3]. This is achieved using antigen-binding variable region (V-region) on IgA to restrict bacterial epitopes preventing adhesion with the surface. IgA also neutralizes microorganisms by limiting motility via nonspecific binding achieved through binding glycans on the constant region of IgA. *Streptococcus mitis* is a commensal microorganism found abundantly in the oral cavity and is also considered an opportunistic organism [4]. When a patient exhibits suppression of immunity, complications of *Streptococcus mitis* infections such as bacteremia and endocarditis may occur. The clinical case discussed in this report is a patient with previously diagnosed IgA MGUS who was found to have *Streptococcus mitis* bacteremia.

### 1.1. Hematologic/Oncologic History

The patient is a 63-year-old Caucasian male with a past medical history of hypertension who was found to have a slightly elevated creatinine from baseline while being worked up for other cardiac issues which prompted serum protein electrophoresis and immunofixation. Bone marrow aspirate and biopsy were obtained which showed approximately 1% plasmacytosis with a slight lambda excess. Studies also showed a monoclonal spike (M spike) of 0.73 g/dL on serum protein electrophoresis. Quantitative immunoglobulins were significant for an elevated IgA of 869 mg/dL and regularly followed up with an oncologist to monitor his MGUS for progression and complications for thirteen years. Laboratory values remained within benign limits, and no interventions were recommended for his disease. A diagnosis of IgA MGUS was confirmed.

### 1.2. Initial Presentation

The patient is a 76-year-old Caucasian male with a past medical history of IgA lambda MGUS diagnosed thirteen years ago, hypertension, hyperlipidemia, systolic heart failure, pacemaker placement, and atrial fibrillation status after ablation procedure 2 weeks prior. This patient presented to the emergency department complaining of persistent cough associated with dyspnea that began following a cardiac ablation for atrial fibrillation. The patient had associated abdominal fullness and fever of 101.8 F at home. Physical examination on admission was significant for blood pressure, 84/48 mmHg; temperature, 99.8°F; heart rate, 70 beats/minute; respiratory rate, 18 breaths per minute; and the patient's oxygen saturation, 98% on room air. General appearance displayed a cooperative and comfortable man who did not appear in any acute distress. The lungs were clear to auscultation bilaterally without the presence of accessory muscle use. Cardiac examination revealed normal heart sounds without any murmurs appreciated. No appreciable skin changes or edema were visualized, and the patient was neurologically intact and oriented to person, place, and time.

The patient clinically declined hours following initial presentation and became altered, with respiratory distress requiring intubation. The patient was admitted to the medical intensive care unit for concerns of septic shock.

### 1.3. Medical Intensive Care Unit (MICU)

The patient was found to have blood cultures positive for *Streptococcus mitis* and subsequently started on vancomycin and ceftriaxone. The patient was also found to have ST elevations with uptrending troponins from 0.7 ng/dL to 258 ng/dL. 2D echocardiogram and transthoracic echocardiogram (TTE) showed no significant changes from baseline cardiac status with an EF of 35%. At this point, the patient became progressively hemodynamically instable and was started on norepinephrine. Hemodynamic instability worsened, and the patient required four vasoactive agents. At this time, the patient's course became complicated by shock liver, thrombocytopenia with a platelet count of 26,000/mm^3^, and declining renal function, BUN and creatinine values of 56 mg/dL and 4.31 mg/dL, respectively. Transesophageal echocardiogram (TEE) was ordered to evaluate for endocarditis. TEE results were consistent with the 2D echo and TTE 2 days prior, and the source remained unclear. The patient continued to clinically decompensate, and power of attorney changed code status to DNR. The patient subsequently passed from mixed cardiogenic and septic shock.

## 2. Discussion

While MGUS is known to be asymptomatic, current research illustrates that may not be the case. The study of [2] demonstrates that MGUS patients have an average 2.2-fold increase in developing infection as compared to a population of patients that do not have the disorder [5]. This study identifies specific infections that are more likely to occur within this patient population including pneumonia, osteomyelitis, septicemia, pyelonephritis, meningitis, cellulitis, and endocarditis [5]. Specifically, this study illustrates a 3.1-fold increase in septicemia in established MGUS patient as compared to a controlled population without the disease [5]. Furthermore, a study published in the *Clinical Interventions in Aging* determined patients with diagnosed MGUS benefit from the 13-valent pneumococcal conjugate vaccine to decrease their risk of future complications due to an underlying immunocompromised nature of the disorder [6].

In regard to the discussed case, this patient with multiple comorbidities controlled by either medications or other interventions with an underlying immunocompromised state is at a higher risk for infection. IgA is the predominant immunoglobin in the mucosa and plays a crucial role in defense of mucosal infections [7]. As a consequence of this disease, decreased levels of polyclonal immunoglobulins in serum and of polyclonal IgA in mucosal secretions may have potentiated corresponding infections. *Streptococcus mitis,* a member of the viridans group of microbes, predominantly inhabits the oral and gastrointestinal mucosa as normal flora and is considered to be an opportunistic pathogen [6]. It is likely that this patient with IgA MGUS and *S. mitis* bacteremia is not a coincidence. It is difficult to determine if more aggressive or tailored treatment may have changed the outcome of this case, although preventative measure should not be ignored in patients with MGUS.

## 3. Conclusion

Given the data supporting the increased infections in patients' with MGUS, prophylactic measures such as 13-valent pneumococcal vaccine could be considered for patients categorized as higher risk. Higher concentrations of M protein are associated with the highest risk of infections in MGUS patients [5]. Given this correlation with M protein and risk of infection, it is reasonable to take extra precautions when a patient with MGUS may be in a physiologically demanding situation, such as postprocedure. Specific coverage for streptococcal species as well as anaerobic coverage for gastrointestinal pathogens may have a benefit in this patient population. Special considerations when treating a patient with MGUS should not be overlooked.

## References

[1] Understanding MGUS (2018). Understanding MGUS and smoldering multiple myeloma. https://myeloma.org/sites/default/files/resource/u-mgus_smm.pdf.

[2] Kaseb H., Babiker H. M. (2020). *Monoclonal Gammopathy of Undetermined Significance*.

[3] Woof J. M., Kerr M. A. (2006). The function of immunoglobulin A in immunity. *The Journal of Pathology*.

[4] Rasmussen L. H., Højholt K., Dargis R. (2017). In silico assessment of virulence factors in strains of Streptococcus oralis and Streptococcus mitis isolated from patients with infective endocarditis. *Journal of Medical Microbiology*.

[5] Kristinsson S. Y., Tang M., Pfeiffer R. M. (2012). Monoclonal gammopathy of undetermined significance and risk of infections: a population-based study. *Haematologica*.

[6] Pasiarski M., Sosnowska-Pasiarska B., Grywalska E. (2019). Immunogenicity and safety of the 13-valent pneumococcal conjugate vaccine in patients with monoclonal gammopathy of undetermined significance–relationship with selected immune and clinical parameters. *Clinical Interventions in Aging*.

[7] Lamm M. E. (1988). The IgA mucosal immune system. *American Journal of Kidney Diseases*.

[8] Shenep J. L. (2000). Viridans-group streptococcal infections in immunocompromised hosts. *International Journal of Antimicrobial Agents*.

[9] Kyle R. A., Buadi F. I., Rajkumar S. V. E. (2011). Management of monoclonal gammopathy of undetermined significance (MGUS) and smoldering multiple myeloma (SMM). *Oncology (Williston Park, NY)*.

[10] Kyle R. A., Durie B. G. M., Rajkumar S. V. (2010). Monoclonal gammopathy of undetermined significance (MGUS) and smoldering (asymptomatic) multiple myeloma: IMWG consensus perspectives risk factors for progression and guidelines for monitoring and management. *Leukemia*.

[11] Zingone A., Kuehl W. M. (2011). Pathogenesis of monoclonal gammopathy of undetermined significance and progression to multiple myeloma. *Seminars in hematology*.

